# Efficacy of 12-week treatment with polyethylene glycol loxenatide in obesity or overweight patients with type 2 diabetes: a multicenter, prospective cohort study based on the flash glucose monitoring system

**DOI:** 10.3389/fendo.2026.1810786

**Published:** 2026-05-19

**Authors:** Liye Zhu, Hongning Li, Yuyang Zhang, Shanshan Tao, Rengna Yan, Yong Luo, Ziyang Shen, Huiqin Li, Xiaocen Kong, Jianhua Ma

**Affiliations:** 1 Department of Endocrinology, Nanjing First Hospital, Nanjing Medical University, Nanjing, China; 2 Department of Endocrinology, Nanjing Lishui People’s Hospital, Zhongda Hospital Lishui Branch, Southeast University, Nanjing, China

**Keywords:** flash glucose monitoring, polyethylene glycol loxenatide, predictors of response, time in range, type 2 diabetes

## Abstract

**Objectives:**

To investigate the factors influencing the glucose-lowering efficacy of polyethylene glycol loxenatide (PEG-Loxe) added to diet, exercise, and existing glucose-lowering therapies in patients with poorly controlled type 2 diabetes mellitus (T2DM) after 12 weeks of treatment.

**Methods:**

This multicenter prospective cohort study with a pre-post intervention design, based on the FreeStyle Libre flash glucose monitoring (FGM) system, enrolled T2DM patients with inadequate glycemic control (HbA_1c >_7.5%) despite stable treatment (diet, exercise, and medication) for at least 8 weeks. PEG-Loxe (0.2 mg once weekly) was added to their original treatment regimen for 12 weeks. Demographic and clinical data were collected. Standardized meal tests were performed before and after treatment to measure glucose, HbA_1c_, insulin, C-peptide, and glucagon. Glycemic profiles, including 24-hour mean blood glucose (MBG), glucose variability (SDBG, CV), time in range (TIR), time above range (TAR), and time below range (TBR), were assessed using the hospital version of the Abbott FreeStyle Libre FGM system.

**Results:**

Of the 500 initially enrolled patients, 246 patients were included in the final analysis. Baseline HbA_1c_ was 8.74% ± 1.07%. After 12 weeks of PEG-Loxe add-on therapy, 39.6% of patients achieved HbA_1c <_6.5%, 63.3% achieved HbA_1c <_7.0%, and 77.12% attained TIR >70%. Significant reductions were observed across multiple parameters. Fasting blood glucose (FBG), postprandial blood glucose (PBG), MBG, TAR, and SDBG were significantly reduced (all P<0.05). Concurrently, TIR, insulin levels, C-peptide levels, and insulin resistance (as measured by HOMA-IR) improved, while 2-hour postprandial glucagon (2h-PGlu) decreased. No significant change in TBR was detected. Correlation analysis revealed that TIR improvement was associated with a shorter diabetes duration and higher baseline HbA_1c_. Furthermore, PEG-Loxe treatment significantly improved cardiometabolic parameters and liver enzymes.

**Conclusions:**

PEG-Loxe significantly improved glycemic control in Chinese patients with T2DM who were overweight or obese. Patients who were younger, had a shorter duration of diabetes, and presented with higher baseline glucose levels appeared to derive greater benefit from the therapy. Beyond glycemic control, PEG-Loxe might also show additional benefits across multiple cardiometabolic indices.

**Clinical trial registration:**

www.clinicaltrials.gov, identifier NCT05611684.

## 1 Introduction

Type 2 diabetes (T2DM) remains a pressing global health crisis, affecting over half a billion adults worldwide and contributing to significant morbidity and mortality (1). The disease pathophysiology is characterized by a complex interplay of insulin resistance and progressive β-cell function decline, making long-term glycemic maintenance challenging (2). Despite the availability of numerous hypoglycemic agents, a substantial proportion of patients fail to achieve recommended glycated hemoglobin A_1c_ (HbA_1c_) targets in real-world clinical practice, highlighting pervasive therapeutic inertia and the urgent need for more effective treatment strategies (3, 4). The advent of glucagon-like peptide-1 receptor agonists (GLP-1 RAs) represents a paradigm shift in T2DM management. This class provides effective glycemic control via glucose-concentration-dependent stimulation of insulin secretion and suppression of glucagon, while also conferring significant weight loss and cardiovascular protection (5).

Polyethylene glycol loxenatide (PEG-Loxe), a pegylated form of the GLP-1 receptor agonist loxenatide, has been designed to enhance the pharmacokinetics and pharmacodynamics of its predecessor. By extending the drug’s half-life, PEG-Loxe aims to provide sustained glycemic control with less frequent dosing, potentially increasing patient adherence and overall treatment effectiveness. Previous randomized controlled trials (RCTs) have demonstrated that PEG-Loxe can effectively lower HbA_1c_ levels and promote weight loss in individuals with T2DM (6–9). Although RCT studies provided high-quality efficacy data, their stringent eligibility criteria created homogeneous cohorts. Subjects enrolled in these RCTs underwent rigorous screening processes, resulting in as study populations rarely reflect real-world patient diversity. This “efficacy-effectiveness gap” is recognized for GLP-1 RAs, underscoring the need for complementary evidence from real-world settings (10).

Real-World Study (RWS) is an observational research method that utilizes data from real-world clinical settings to evaluate the effectiveness and safety of interventions in routine practice. RWS and RCT are complementary relationships, together forming a complete ecosystem of evidence (11). Furthermore, clinical observation reveals significant inter-individual variability in patient response to GLP-1 RAs. Therefore, it is of great significance for clinical practice to carry out a real world study on Chinese population and observe the effect of PEG-Loxe on Chinese T2DM patients. This multicenter, prospective cohort study aimed to evaluate the practical effectiveness of a 12-week PEG-Loxe add-on therapy in adults with inadequately controlled T2DM. We sought to comprehensively assess its impact on glycemic parameters (including HbA_1c_ and Time-in-Range), β-cell function, and a range of cardiometabolic indices. More importantly, we endeavored to identify key clinical predictors of treatment response, with specific focus on diabetes duration and baseline HbA_1c_ levels. These findings are expected to provide valuable insights into how different factors can influence treatment efficacy and help guide personalized treatment approaches for patients with T2DM.

## 2 Materials and methods

### 2.1 Study population

This study was conducted at 17 hospitals in Jiangsu Province, China. It was approved by the Ethics Committee of Nanjing First Hospital (KY20220825-03). All procedures followed were in accordance with the Helsinki Declaration of 1964, as revised in 2013. Informed consent was obtained from all patients for inclusion in this study. It was registered at ClinicalTrial.gov with registration number NCT05611684.

The patient inclusion criteria were as follows: (1) Aged 18–75 years with a body mass index (BMI) ≥24 kg/m2 and diagnosis of T2DM (meeting the WHO1999 diagnostic criteria); (2) Insufficient glycemic control (HbA_1c_ ≥7.5%) for all drug doses were stable for more than 2 months; (3) Subjects were able and willing to monitor peripheral blood sugar and regularity of diet and exercise; (4) Volunteered to participate and provided signed informed consent prior to the trial. The exclusion criteria were as follows: (1) T1DM; (2) Hepatic and renal dysfunctions: alanine transferase (ALT) higher than the 2.5 times the upper limit of normal or serum creatinine (SCr) higher than the 1.3 times the upper limit of normal; (3) Use of any of the following medications or therapies within one year prior to screening: glucagon-like peptide-1 receptor agonists (GLP-1RAs), GLP-1 analogues, dipeptidyl peptidase-4 inhibitors (DPP-4 inhibitors), or any other incretin mimetics. (4) History of acute or chronic pancreatitis; Personal or family history of medullary thyroid carcinoma (MTC), Multiple Endocrine Neoplasia type 2A (MEN 2A) or type 2B (MEN 2B) syndromes. (5) Use of weight-control medications (e.g., anti-obesity pharmacotherapy) within 2 months prior to screening; History of weight-altering surgeries (e.g., bariatric surgery); Current participation in a weight-loss program (non-maintenance phase). (6) Prolonged use (≥7 consecutive days) of corticosteroids via intravenous, oral, or intra-articular routes within 2 months prior to screening. (7) Presence of clinically significant gastric emptying abnormalities. (8) acute infections or acute complications in the past 6 months. (9) History of malignancies (treated or untreated) in any organ system within 5 years prior to screening. (10) History of coronary angioplasty, decompensated heart failure (New York Heart Association Class III or IV), stroke, transient ischemic attack, unstable angina, myocardial infarction, or clinically significant cardiac arrhythmias within 6 months prior to screening.

### 2.2 Study design

**Figure 1:**
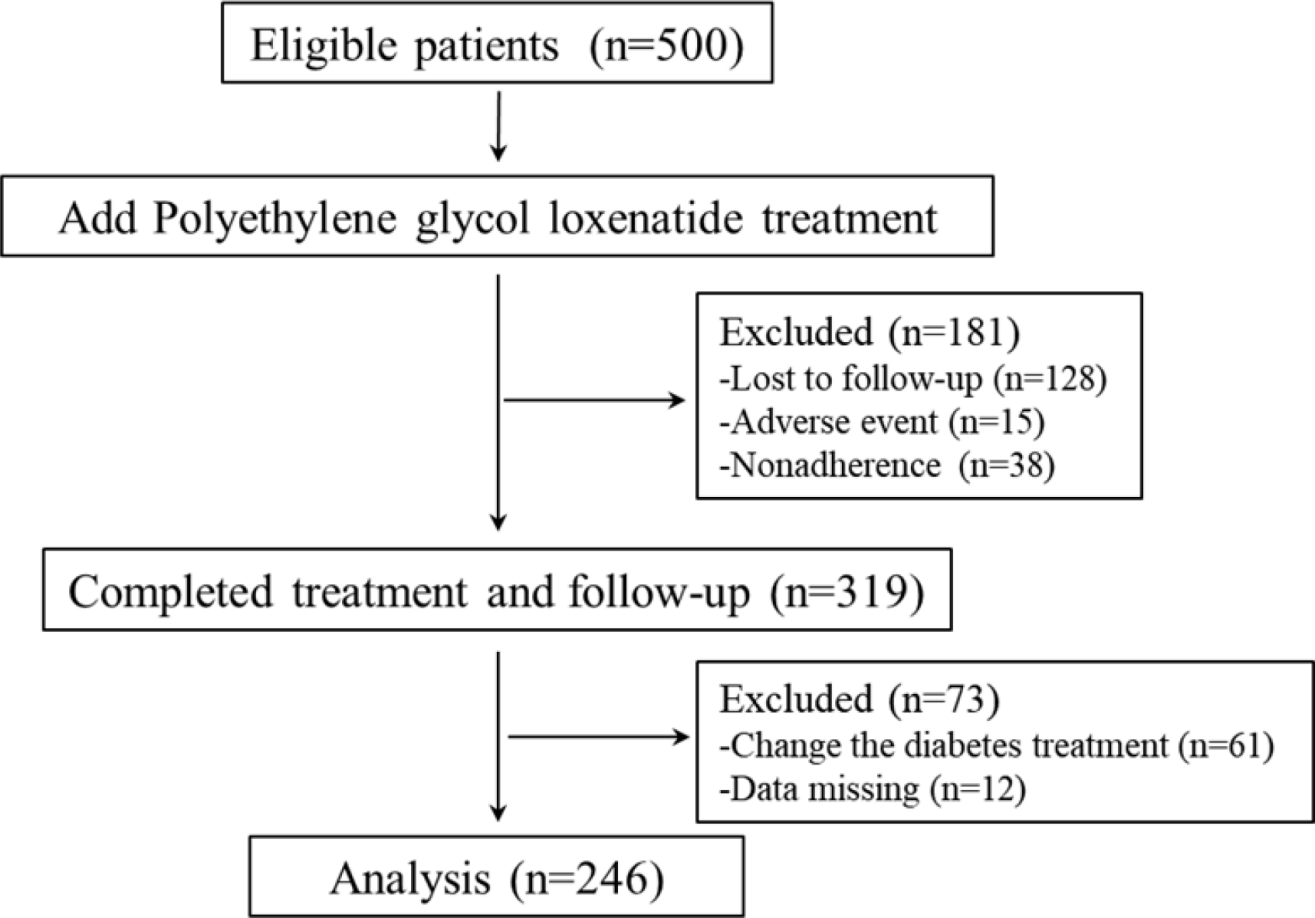
Study design and the flowchart of patients in the study.

In this study, 500 eligible obesity or overweight patients with T2DM were screened to receive add-on abdominal subcutaneous injections of PEG-Loxe (0.2mg Once a week) for 12 weeks. 128 patients lost to follow-up, 15 patients discontinued due to gastrointestinal reactions, 38 patients refused medication. The study design and flowchart of patients in this trial were shown in **Figure 1**.

The study lasted for 13 weeks. All patients received a standardized diet and were also required to refrain from both structured and recreational physical activities. There was no change in the type or dosage of medication taken by the patients before enrollment and during this study. All subjects received oral standard meal tolerance test (MTT) and FGM at baseline and week 11 of the treatment. The subjects were required to visit the local study center every four weeks.

### 2.3 Clinical and laboratory examination

Patient data, including demographic characteristics, lifestyle habits, medical history and medication use, were obtained using a standard questionnaire. Height and body weight were measured using a digital scale, and BMI was calculated as weight divided by height squared (kg/m^2^). Blood samples were collected after the patients fasted overnight (≥ 8 hours). Biochemical parameters, including blood glucose (BG), ALT, aspartate aminotransferase (AST), total cholesterol (TC), triglyceride (TG), low density lipoprotein (LDL), high density lipoprotein (HDL), SCr and uric acid were measured using routine laboratory methods using a HITACHI 7600 device (HITACHI, Tokyo, Japan). Levels of insulin were measured with a radioimmunoassay kit. C-peptide (C-p) were assessed with chemiluminescent microparticle immunoassay (Architect system, USA). The homeostasis model assessment for insulin resistance (HOMA-IR), β cell function (HOMA-β), insulinogenic Index (IGI), whole-body insulin sensitivity index (Matsuda-index), and insulin secretion-sensitivity index-2 (ISSI-2) were assessed through previously published procedures (12).

### 2.4 The FGM system

The FreeStyle Libre professional-mode FGM system (Abbott Diabetes Care, UK) has a disposable sensor, a handheld reader, and associated software. The sensor was applied at the clinic to the upper arm of the participants for 7 days and recorded interstitial glucose concentrations every 15 min. The glucose data were not available to the patients. After completion of the FGM intervention, the glucose data were downloaded. We analyzed the FGM data from day 2 to day 6. The following glycemic variability indices obtained from the FGM were calculated: 24-h mean blood glucose (24h MBG) (defined as the average glucose of 96 measurements equally spaced in time), mean amplitude of glycemic excursion (MAGE), standard deviation of mean glucose (SDBG), and coefficient of variation (CV %). Time in range (TIR, glucose level 3.9-10.0 mmol/L), time above range (TAR, glucose level > 10.0 mmol/L), and time below range (TBR, glucose level < 3.9mmol/L) were also calculated. The incremental area under the curve (AUC) of the glucose level > 10.0 mmol/L and area over the curve (AOC) of a glucose level < 3.9 mmol/L were calculated using the trapezoid rule.

### 2.5 Statistical analysis

All statistical analyses were performed using SPSS 22.0 (SPSS Inc., USA) and GraphPad Prism 6.0 (GraphPad Inc., USA). Data are described as mean ± standard deviation (SD) for normally distributed continuous variables and median with interquartile range for non-normally distributed continuous variables. Categorical data are presented as frequency. Comparisons between the pre- and post-treatment levels of parameters were made using paired t-test for normally distributed continuous variables and Wilcoxon matched‐pairs test for non-normally distributed continuous parameters. Linear correlation analyses were used to evaluate the relationship between ▵TIR and other variables. Multiple stepwise regression analyses were used to evaluate the relationship between ▵TIR and variables. Differences in glycemic variation characteristics between the two groups were assessed using the Wilcoxon test. A *P*-value of less than 0.05 was considered statistically significant.

## 3 Results

### 3.1 Baseline characteristics

A total of 500 patients were recruited, and 319 patients with T2DM successfully completed this study. After excluding patients who adjusted to original regimens during the study or had missing data, 246 patients (159 men and 87 women, with a mean age of 53.76 ± 10.86 years) were included in the final analysis. Their diabetic duration was 4 years and 39% of patients received metformin. The Clinical characteristics of the subjects are described in **Table 1**.

**Table 1:** Baseline characteristics of patients.

Characteristics	Baseline
Sex (male/female)	159/87
Age (years)	53.76 ± 10.86
Diabetic duration (years)	4 (1, 10)
Use of metformin (n(%))	91 (36.99%)
Use of sulphonylurea (n(%))	48 (19.51%)
Use of SGLT-2 inhibitors (n(%))	40 (16.26%)
Use of α-glucosidase inhibitors (n(%))	32 (13.01%)
Use of TZD (n(%))	16 (6.5%)
Use of insulin (n(%))	39 (15.85%)

SGLT-2, sodium-dependent glucose transporter 2; TZD, thiazolidinedione.

Baseline characteristics were generally well balanced between included (n=246) and excluded (n=254) patients, except that excluded patients were significantly younger (48.32 vs 53.76 years, *P* = 0.001). No other significant differences were observed for sex, diabetic duration, weight, BMI, waist circumference, or glycemic parameters (all *P*>0.05; **Supplementary Table 1**).

### 3.2 Blood glucose levels and islet function

**Figure 2:**
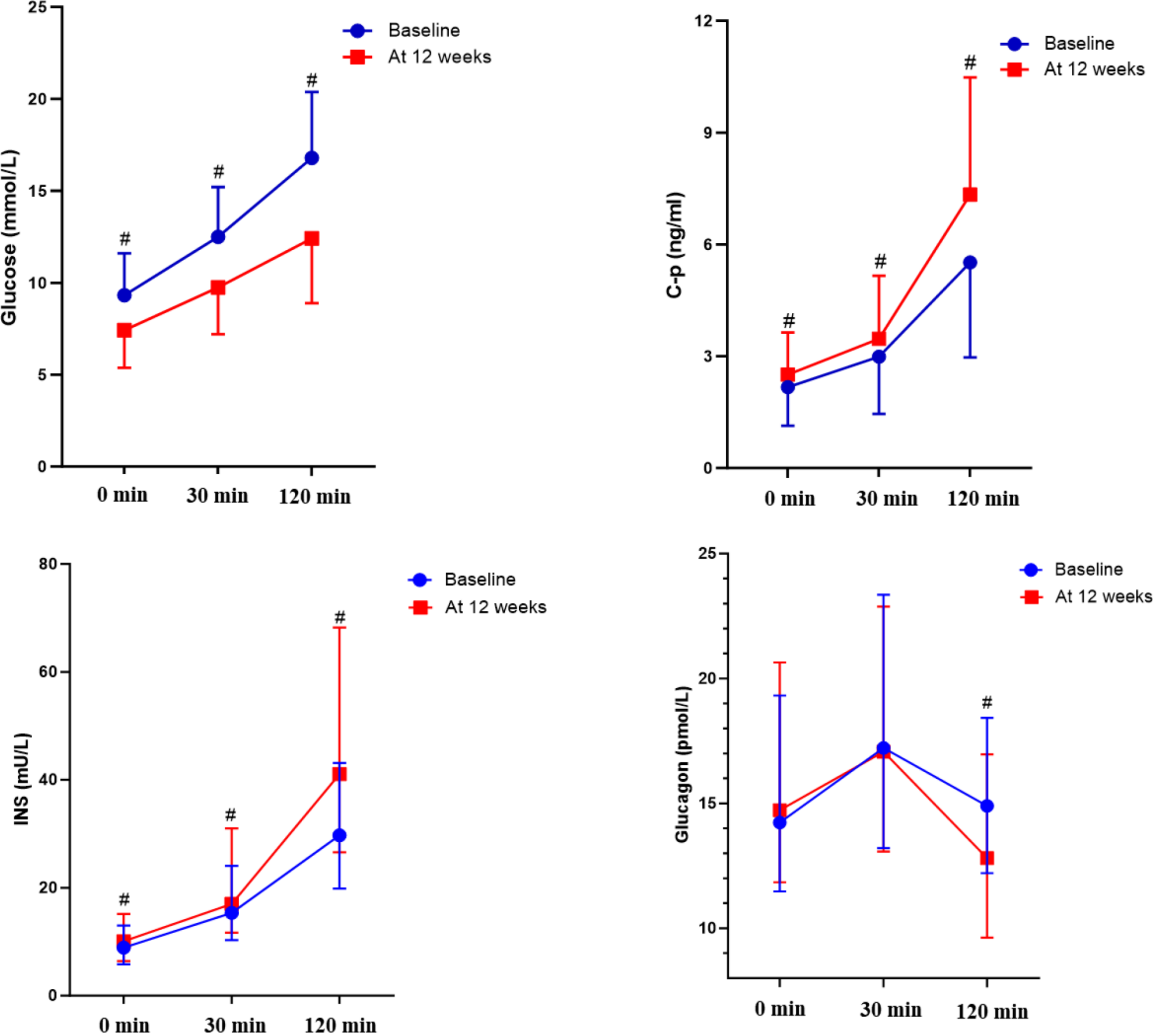
Changes of index for glucose, insulin secretion and glucagon from baseline to week 12. ^#^*p* < 0.001.

PEG-Loxe (0.2mg once a week) treatment 12 weeks could significantly improve the glycemic control. As presented in **Table 2**, the HbA_1c_ at 12 weeks was significantly lower than baseline (6.86 ± 0.85 vs. 8.74 ± 1.07%, *P* < 0.001). After 12 weeks of PEG-Loxe adding-on therapy: 39.6% of patients achieved HbA_1c <_6.5%, 63.3% achieved HbA_1c <_7.0%. All of FBG, 30min and 120min-PBG decreased significantly in both groups (**Figure 2A**). In terms of C-peptide and insulin levels (**Figures 2B, C**), PEG-Loxe treatment had effects on increasing fasting insulin and C-peptide levels, 30min and 120min postprandial insulin and C-peptide levels. Furthermore, PEG-Loxe treatment could reduce 120min postprandial glucagon level (**Figure 2D**). After 12 weeks of PEG-Loxe therapy, both HOMA-β, IGI and ISSI-2 levels were significantly higher than baseline (*P* < 0.01). Meanwhile, HOMA-IR decreased significantly (P<0.001) (**Table 2**).

**Table 2:** Characteristics of glycemic control before and after polyethylene glycol loxenatide treatment.

Characteristics	Baseline	At 12 weeks	△value	*P*
HbA_1c_ (%)	8.74 ± 1.07	6.86 ± 0.85	-1.88 ± 1.01	<0.001
FBG (mmol/L)	9.32 ± 2.28	7.42 ± 2.04	-1.98 ± 2.14	<0.001
FC-p (ng/ml)	2.17 ± 1.03	2.51 ± 1.13	0.25 (-0.02, 0.65)	<0.001
FINS (mU/L)	8.91 (5.81, 12.97)	10.06 (6.38, 15.13)	0.83 (-0.96, 3.55)	<0.001
HOMA-β	32.31 (22.16, 47.66)	56.73 (39.12, 91.76)	23.82 (12.46, 48.88)	<0.001
HOMA-IR	3.85 (2.49, 6.43)	3.24 (1.95, 5.48)	-0.51 (-1.78, 0.40)	<0.001
IGI	0.88 (0.48, 1.69)	3.24 (1.41, 7.41)	2.32 (0.46, 5.88)	<0.001
Matsuda-index	70.80 (43.14, 101.42)	76.01 (47.88, 119.21)	4.56 (-11.94, 25.17)	0.080
ISSI-2	19.95 (12.74, 26.35)	37.65 (20.85, 57.04)	15.20 (2.99, 33.89)	<0.001

FC-p, fasting C-peptide; FINS, fasting insuline; HOMA-β, the homeostasis model assessment for β cell function; HOMA-IR, the homeostasis model assessment for insulin resistance; IGI, insulinogenic index; Matsuda-index, whole-body insulin sensitivity index; ISSI-2, insulin secretion-sensitivity index-2.

### 3.3 FGM glucose profile

**Table 3** showed the glucose fluctuation variables measured using an FGM before and after PEG-Loxe treatment 12 weeks. The 24h MBG was significant lower at PEG-Loxe treatment 12 weeks than baseline (7.81 ± 1.61 vs. 10.14 ± 2.23, *P* < 0.001). The TIR 3.9-10mmol/L at 12 weeks was significantly higher than baseline (82.59 ± 18.24 vs. 55.21 ± 27.90%, *P* < 0.001). Meanwhile, 77.12% patients attained TIR >70% after 12 weeks of PEG-Loxe add-on therapy. The TAR>10mmol/L at 12 weeks was significantly lower than baseline (*P* < 0.001). Difference was also seen in the SDBG (1.77 ± 0.78 vs. 2.32 ± 0.81, *P* < 0.001). There was no difference in the parameters of CV% and TBR before and after PEG-Loxe treatment 12 weeks.

**Table 3:** Characteristics of daily glycemic control before and after polyethylene glycol loxenatide treatment.

Characteristics	Baseline	At 12 weeks	▵value	*P*
MBG (mmol/L)	10.14 ± 2.23	7.81 ± 1.61	-2.33 ± 2.01	<0.001
SDBG	2.32 ± 0.81	1.77 ± 0.78	-0.54 ± 1.00	<0.001
CV (%)	23.21 ± 7.54	22.33 ± 7.62	-0.89 ± 9.19	0.295
TIR (%)	55.21 ± 27.90	82.59 ± 18.24	27.38 ± 24.99	<0.001
TAR (%)	38.02 (22.83, 67.19)	10.07 (0.87, 26.74)	-27.94 ± 24.40	<0.001
TBR (%)	0.00 (0.00, 0.00)	0.00 (0.00, 0.00)	0.00 (0.00, 0.00)	0.378

MBG, 24-h mean blood glucose; SDBG, standard deviation of mean glucose; CV, coefficient of variation; TIR, time in range (glucose level 3.9-10.0 mmol/L); TAR, time above range (glucose level > 10.0 mmol/L); TBR, time below range (glucose level < 3.9mmol/L).

**Figure 3:**
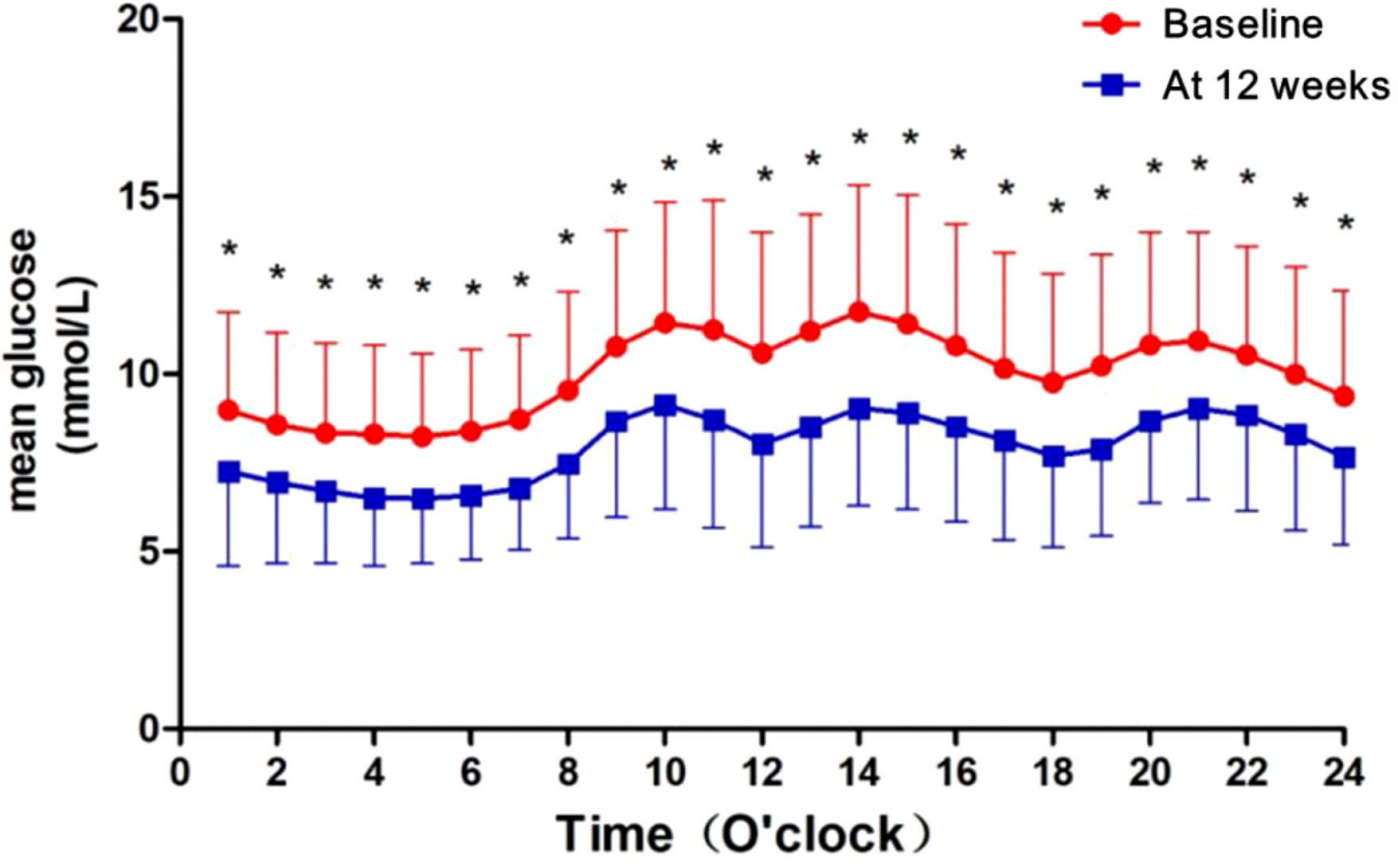
The 24hr FGM glucose fluctuation profile before and after Polyethylene glycol loxenatide treatment. *p<0.001.

The average blood glucose concentration per hour measured using the FGM in patients before and after administration of PEG-Loxe was shown in **Figure 3**. We observed that patients adding-on PEG-Loxe treatment 12 weeks had significantly lower hourly mean glucose levels.

### 3.4 Factors influencing TIR variation

The relationship between ▵TIR and related clinical variables in all of the subjects was examined through linear correlation analyses (**Table 4**). ▵TIR was correlated positively with HbA_1c_ (Baseline), FBG (Baseline), 2h-PBG (Baseline), MBG (Baseline) and ▵ISSI-2. In contrast, ▵TIR was correlated negatively with duration of T2DM, ▵HbA_1c_, ▵FBG, ▵2h-PBG, ▵MBG, HOMA-β and IGI. The multiple regression analysis subsequently revealed a significant negative correlation between the duration of T2DM and ▵TIR (β = -0.193, p=0.042), while a positive correlation was observed between HbA_1c_ (Baseline) and ▵TIR (β=0.261, p=0.006) (**Table 5**). Despite modest overall fit (R^2^ = 0.114), both variables are statistically significant (p < 0.05), highlighting their independent contributions. This suggests that prolonged diabetes duration potentially limits glycemic control improvement, whereas patients with elevated baseline HbA_1c_ levels exhibit more significant therapeutic gains. However, the model explained only 11.4% of the variance (R² = 0.114, adjusted R² = 0.096), indicating that the vast majority (88.6%) of variability in TIR improvement remains unexplained by these two variables. Therefore, while duration of T2DM and baseline HbA_1c_ show statistically significant associations with ΔTIR, their explanatory power is very limited.

**Table 4:** Linear correlation analysis of ▵TIR with other variables.

Variables	*r*	*P*
age	-0.123	0.192
Duration of T2DM	-0.203	0.029
HbA_1c_ (Baseline)	0.263	0.005
▵HbA_1c_	-0.203	0.029
FBG (Baseline)	0.309	<0.001
▵FBG	-0.263	0.04
2h-PBG (Baseline)	0.267	0.004
▵2h-PBG	-0.229	<0.001
MBG (Baseline)	0.753	<0.001
▵MBG	-0.823	<0.001
HOMA-IR	-0.027	0.784
HOMA-β	-0.194	0.045
IGI	-0.194	0.048
Matsuda-Index	0.094	0.331
ISSI-2	-0.039	0.689
▵ISSI-2	0.208	0.031

*r* correlation coefficient.

FBG, fasting blood glucose; PBG, postprandial blood glucose; MBG, 24-h mean blood glucose; HOMA-IR, the homeostasis model assessment for insulin resistance; HOMA-β, the homeostasis model assessment for β cell function; IGI, insulinogenic index; Matsuda-index, whole-body insulin sensitivity index; ISSI-2, insulin secretion-sensitivity index-2.

**Table 5:** Multiple stepwise regression analysis of the relationship between ▵TIR and variables.

Variables	β	t-value	P-value
Duration of T2DM	-0.193	-2.063	0.042
HbA_1c_ (Baseline)	0.261	2.791	0.006

▵TIR is regarded as the dependent variable, and independent variables included duration of T2DM and HbA_1c_ (Baseline).

R2 = 0.114, adjustedR2 = 0.096.

A comparative analysis was performed to examine the characteristics of glycemic parameter changes in subjects categorized according to their baseline HbA_1c_ levels (≥9% vs. <9%, **Table 6**). The high baseline HbA_1c_ group (≥9%) demonstrated significantly greater improvements across multiple glycemic parameters compared to the lower baseline HbA_1c_ group (<9%), including lower ▵MBG, higher ▵TIR, lower ▵TAR and ▵HbA_1c_ (P<0.05). No significant between-group differences were observed for glucose variability (▵SDBG, p=0.442; ▵CV, P = 0.798) and hypoglycemia metrics (▵TBR, P = 0.237).

**Table 6:** Glycemic variation characteristics: baseline HbA_1c_ ≥9% vs. <9%.

Characteristics	HbA_1c_≥9% (N = 98)	HbA_1c_<9% (N = 148)	P-value
▵MBG	-2.70 (-4.68, -0.99)	-1.72 (-2.79, -0.92)	0.013
▵SDBG	-0.58 (-1.28, -0.03)	-0.43 (-1.01, 0.22)	0.442
▵CV	-0.32 (-5.98, 4.82)	-0.96 (-8.00, 5.57)	0.798
▵TIR	37.85 (13.71, 51.04)	20.48 (7.03, 35.68)	0.004
▵TBR	0.00 (0.00, 0.00)	0.00 (0.00, 0.00)	0.237
▵TAR	-37.85 (-51.04, -13.71)	-20.48 (-35.68, 8.15)	0.006
▵HbA_1c_	-2.85 (-3.70, -2.20)	-1.5 (-2.00, -0.90)	<0.001

MBG, 24-h mean blood glucose; SDBG, standard deviation of mean glucose; CV, coefficient of variation; TIR, time in range (glucose level 3.9-10.0 mmol/L); TAR, time above range (glucose level > 10.0 mmol/L); TBR, time below range (glucose level < 3.9mmol/L).

Furthermore, we compared glycemic control improvements between patients with longer (≥5 years) versus shorter (<5 years) diabetes duration (**Table 7**). Patients with shorter diabetes duration demonstrated significantly greater improvements across multiple glycemic parameters: achieved 0.81 mmol/L greater reduction in MBG (p=0.004); showed 8.69% larger increase in TIR (p=0.046); had 8.68% greater reduction in hyperglycemia exposure (p=0.045); exhibited 0.7% greater HbA_1c_ reduction (p<0.001). The shorter duration group also showed substantially better preservation of β-cell function: 8.77-point greater improvement in ISSI-2 (p=0.009). This represents an 82% greater improvement compared to the longer duration group. There were no significant differences in the glucose variability measures (SDBG, CV) and hypoglycemia incidence (TBR).

**Table 7:** Glycemic variation characteristics: duration of T2DM ≥ 5years vs. < 5years.

Characteristics	≥ 5 years (N = 106)	< 5years (N = 140)	P-value
▵MBG	-1.60 (-2.55, -0.59)	-2.41 (-4.15, -1.03)	0.004
▵SDBG	-0.40 (-0.93, 0.22)	-0.56 (-1.26, 0.12)	0.233
▵CV	-1.95 (-6.87, 4.22)	0.11 (-7.04, 5.70)	0.835
▵TIR	19.79 (4.17, 35.42)	28.48 (11.46, 47.40)	0.046
▵TBR	0.00 (0.00, 0.00)	0.00 (0.00, 0.00)	0.743
▵TAR	-19.79 (-35.42, -7.64)	-28.47 (-47.40, -11.72)	0.045
▵HbA_1c_	-1.60 (-2.30, -0.90)	-2.30 (-2.90, -1.50)	<0.001
▵ISSI2	10.65 (0.46, 28.40)	19.42 (4.79, 40.58)	0.009

MBG, 24-h mean blood glucose; SDBG, standard deviation of mean glucose; CV, coefficient of variation; TIR, time in range (glucose level 3.9-10.0 mmol/L); TAR, time above range (glucose level > 10.0 mmol/L); TBR, time below range (glucose level < 3.9mmol/L); ISSI-2, insulin secretion-sensitivity index-2.

### 3.5 Cardiometabolic parameter changes

After 12 weeks of PEG-Loxe treatment, body weight showed reduction (74.81 ± 12.74 vs. 75.92 ± 12.77 kg, P<0.001). Similarly, BMI values at 12 weeks were lower than baseline measurements (26.9 ± 3.28 vs. 27.4 ± 3.46 kg/m², P<0.001). Cardiovascular improvements included: WC (-1.2cm, P = 0.014), WHtR (-0.01, P = 0.011), SBP (-3.9mmHg, P<0.001), and DBP (-1.8mmHg, P = 0.002). PEG-Loxe treatment improved liver enzymes (ALT -7.7%, AST -6.8%) and lipid profile (LDL -4.2%, HDL + 4.5%) after 12 weeks (all P<0.05). While these changes reached statistical significance, the effect sizes were uniformly small, and the absolute magnitudes are modest. No significant changes were observed in heart rate (P = 0.095), total cholesterol (P = 0.170), or triglyceride levels (P = 0.072) following the intervention (**Table 8**).

**Table 8:** Characteristics of control before and after polyethylene glycol loxenatide treatment.

Characteristics	Baseline	At 12 weeks	▵value	*P*
Weight (kg)	75.92 ± 12.77	74.81 ± 12.74	-0.7 (-2.6, 0.2)	<0.001
BMI (kg/m^2^)	27.4 ± 3.46	26.9 ± 3.28	-0.5 (-0.1, 0.0)	<0.001
WC (cm)	95.5 ± 9.34	94.3 ± 11.01	-1.2 (-3.0, 0.0)	0.014
WHtR	0.573 ± 0.056	0.565 ± 0.063	-0.01 (-0.02, 0.00)	0.011
SBP (mmHg)	130.1 ± 13.71	126.2 ± 12.27	-3.9 (-10.00, 0.00)	<0.001
DBP (mmHg)	82.6 ± 9.95	80.8 ± 9.69	-1.8 (-6.00, 1.00)	0.002
HR (bpm)	80.1 ± 10.18	79.2 ± 9.96	-0.8 (-5.00, 1.00)	0.095
ALT (U/L)	27.80 (19.00, 44.5)	25.65 (18.55, 35.07)	0.00 (-13.05, 11.77)	<0.001
AST (U/L)	22.00 (17.30, 29.40)	20.50 (16.00, 26.00)	0.00 (-6.67, 5.00)	<0.001
BUN (mmol/l)	5.48 ± 1.58	5.52 ± 1.50	0.30 (-0.57, 1.54)	0.714
Scr (ummol/L)	63.99 ± 16.12	65.74 ± 16.09	2.70 (-2.68, 11.56)	0.008
TC (mmol/L)	4.74 ± 1.10	4.64 ± 1.13	-0.18 (-0.65, 0.39)	0.170
TG (mmol/L)	2.17 ± 1.36	2.01 ± 1.28	-1.00 (-0.59, 0.23)	0.072
LDL (mmol/L)	2.84 ± 0.89	2.72 ± 0.85	**-**0.10 (-0.51, 0.35)	0.024
HDL (mmol/L)	1.11 ± 0.24	1.16 ± 0.25	0.05 (-0.07, 0.17)	<0.001

BMI, body mass index; WC, waist circumference; HC, hip circumference; WHtR, waist-to-height ratio; SBP, systolic blood pressure; DBP, diastolic blood pressure; HR, heart rate; ALT, alanine aminotransferase; AST, aspartate aminotransferase; BUN, blood urea nitrogen; Scr, serum creatinine; TC, total cholesterol; TG, triglyceride; HDL, low density lipoprotein; LDL, high density lipoprotein.

## 4 Discussion

This multicenter, prospective cohort study demonstrated the real-world effectiveness of 12-week PEG-Loxe add-on therapy (0.2 mg/week) in patients with inadequately controlled T2DM (mean baseline HbA_1c_ 8.74%). The findings revealed significant glycemic improvements and elucidate key predictors of treatment response. PEG-Loxe add-on therapy elicited multifaceted improvements in glycemic control, β-cell function, and a rgnge of cardiometabolic parameters in T2DM patients. PEG-Loxe is one of the choices for the Early T2DM (duration <5 years) with high baseline HbA_1c_ (≥9%) and patients needing weight loss and CVD risk reduction.

Our core finding was that PEG-Loxe induced a clinically and statistically significant mean reduction in HbA_1c_ of 1.8% from a high baseline of 8.74%. This robust glycemic lowering is complemented by a remarkable improvement in the quality of glycemic control, as evidenced by a 27.4% increase in TIR, enabling 77.12% of patients to achieve the internationally recommended target of TIR >70% (13). The concurrent reduction in glycemic variability (SDBG) and TAR, without an increase in TBR, underscores PEG-Loxe’s ability to stabilize glucose levels effectively without elevating hypoglycemia risk. These glycemic benefits are underpinned by direct effects on pancreatic islet physiology. We observed significant improvements in surrogates of β-cell function (HOMA-β, IGI, ISSI-2) and insulin sensitivity (HOMA-IR), coupled with a potent suppression of postprandial glucagon. This confirmed that PEG-Loxe operated via the established multi-faceted mechanism of the GLP-1 receptor agonist class, which included augmenting glucose-dependent insulin secretion and suppressing inappropriately elevated glucagon levels (14).

The significant improvement in TIR positions PEG-Loxe as a highly effective agent for enhancing the quality of glycemic control. This is corroborated by a recent multicenter retrospective study which found that once-weekly GLP-1 RAs, specifically PEG-Loxe and semaglutide, were significantly more effective at improving TIR than the once-daily agent liraglutide, suggesting that the prolonged pharmacokinetic profile of weekly formulations translates into smoother glycemic control (15). When contextualized within the therapeutic landscape, the glycemic potency of PEG-Loxe observed in our study appears robust. The mean HbA_1c_ reduction of -1.8% is numerically greater than that reported in several real-world studies of other once-weekly GLP-1 RAs. For instance, the SURE program reported mean HbA_1c_ reductions for semaglutide ranging from -1.2% to -1.5% (16, 17), and a large Korean real-world study of dulaglutide showed a mean reduction of -0.95% (18). However, this comparison must be interpreted with caution, as our cohort’s high baseline HbA_1c_ (8.74%) is a key determinant of the magnitude of reduction. It is well-established that higher baseline HbA_1c_ predicts a greater therapeutic response, a finding consistent across multiple GLP-1 RA studies (5).

Our study’s finding that shorter diabetes duration (<5 years) and higher baseline HbA_1c_ (≥9%) are independent predictors of greater improvement in both HbA_1c_ and TIR provides strong real-world validation for the “early, intensive treatment” paradigm. This aligns with evidence for other GLP-1 RAs, where patients with shorter disease duration and higher baseline glucose levels derive the most benefit, likely due to better-preserved β-cell function (19). The superior improvement in ISSI-2 in our shorter-duration cohort provides physiological support for this principle. This analysis provides quantitative evidence supporting current guidelines emphasizing early, aggressive glycemic control while highlighting the diminishing returns of delayed intervention. The findings underscore the need for timely treatment intensification in type 2 diabetes management.

Furthermore, consistent with the known pleiotropic effects of the GLP-1 RA class, PEG-Loxe conferred metabolic advantages beyond glycemic control. Patients experienced reductions in body weight, blood pressure, and atherogenic lipids (LDL-C), alongside an increase in HDL-C. Notably, we observed a decrease in liver enzymes (ALT and AST), providing real-world evidence that PEG-Loxe may ameliorate hepatic steatosis, a benefit increasingly recognized for this drug class in the context of metabolic dysfunction-associated steatotic liver disease (MASLD) (20). These comprehensive improvements in cardiovascular risk factors are crucial for the holistic management of T2DM (21). Both domestic and international guidelines recommend the use of GLP-1RA with evidence of cardiovascular benefits in type 2 diabetes patients with cardiovascular risk factors (22). FLYING trial found that compared with the use of non-incretin anti-diabetes drugs, PEG-Loxe reduced the major adverse cardiovascular event (MACE) risk of type 2 diabetes patients with a history of cardiovascular disease or cardiovascular risk factors by 32% (23). The results suggested that the cardiovascular benefits of GLP-1RA might be drug-class effects.

While the observed improvements in weight, blood pressure, and lipids were statistically significant, their clinical relevance required careful interpretation. A weight loss of 0.7 kg over 12 weeks was substantially less than the 5–10% body weight reduction typically associated with cardiometabolic benefits from GLP-1 receptor agonists in large cardiovascular outcomes trials. The modest changes observed here might reflect the relatively short treatment duration, the lack of a structured lifestyle intervention, or the specific patient population. Similarly, systolic BP reductions of 3.9 mmHg, while statistically significant, are below the thresholds typically considered clinically meaningful (≥5 mmHg). The lipid changes were also modest and unlikely to translate into substantial cardiovascular risk reduction over the short term. Therefore, while these changes are directionally favorable, we could not claim clinically meaningful cardiometabolic benefits from this 12-week study. The primary value of these findings was hypothesis-generating for future longer-term studies.

Several limitations of this study must be taken into account. First, the single-arm, non-comparative observational design means that the observed improvements cannot be unequivocally attributed to PEG-Loxe therapy. Meanwhile, this study precludes definitive conclusions about the relative efficacy of PEG-Loxe compared to other therapies. Without an active comparator or placebo group, the observed effects cannot be unequivocally attributed to the drug alone, as confounding factors such as the Hawthorne effect may have contributed. Second, the study is subject to a significant risk of selection bias. The exclusion of 30.8% of the initially enrolled patients due to loss to follow-up or treatment discontinuation is a major concern. High attrition can lead to an “enriched responder” cohort, as patients who tolerate the medication well and experience a positive response are more likely to complete the study. This may result in an overestimation of the treatment’s mean efficacy and tolerability in a broader, unselected patient population. Third, the significant age difference between included and excluded patients indicates attrition bias, and the final sample likely constitutes an enriched responder population (older, with better follow-up adherence). However, GI adverse events were not a major cause of dropout (only 15 patients, 3.0% of enrolled). Fourth, the 12-week observation period is relatively short and insufficient to assess the long-term durability of the observed benefits or to evaluate long-term safety. The sustainability of glycemic and weight improvements with GLP-1 RAs is typically confirmed in studies of at least 24 to 52 weeks.

To address these limitations and definitively establish the position of PEG-Loxe within the GLP-1 RA class, a long-term, head-to-head randomized controlled trial is imperative. The ideal design would be a multicenter, active-controlled study of at least 52 weeks, comparing PEG-Loxe directly with a potent agent such as once-weekly semaglutide. Co-primary endpoints should include the change in HbA_1c_ and TIR, with key secondary endpoints assessing weight loss, cardiovascular risk markers, and long-term safety. Beyond a confirmatory RCT, future research should explore the long-term cardiovascular and renal protective effects of PEG-Loxe in large-scale observational studies using administrative health databases.

## 5 Conclusion

PEG-Loxe significantly improved blood glucose control in T2DM patients through a triple mechanism of “enhanced insulin secretion, glucagon inhibition and insulin sensitization”, and exhibited additional benefits in multiple metabolic fields such as weight, blood pressure, and blood lipids. Patients with a disease course of less than 5 years and baseline HbA_1c_ ≥ 9% benefit the most significantly, providing new evidence for the “early intensive treatment” strategy. However, due to the absence of a control group, no causal inferences can be made regarding the efficacy of PEG-Loxe. Future randomized controlled trials with longer follow-up are needed to establish the causal effects of PEG-Loxe on glycemic control and cardiometabolic outcomes in patients with type 2 diabetes.

## Data Availability

The raw data supporting the conclusions of this article will be made available by the authors, without undue reservation.

## References

[1] Sun H Saeedi P Karuranga S Pinkepank M Ogurtsova K Duncan BB . IDF Diabetes Atlas: Global, regional and country-level diabetes prevalence estimates for 2021 and projections for 2045. Diabetes Res Clin Pract. (2022) 183:109119. doi: 10.1016/j.diabres.2021.109119. PMID: 34879977 PMC11057359

[2] Kalyani RR Neumiller JJ Maruthur NM Wexler DJ . Diagnosis and treatment of type 2 diabetes in adults: A review. Jama. (2025) 334:984–1002. doi: 10.1001/jama.2025.5956. PMID: 40549398 PMC13014273

[3] Wang L Peng W Zhao Z Zhang M Shi Z Song Z . Prevalence and treatment of diabetes in China, 2013-2018. JAMA. (2021) 326:2498–506. doi: 10.1001/jama.2021.22208. PMID: 34962526 PMC8715349

[4] Xu Y Lu J Li M Wang T Wang K Cao Q . Diabetes in China part 2: prevention, challenges, and progress. Lancet Public Health. (2024) 9:e1098–104. doi: 10.1016/s2468-2667(24)00251-2. PMID: 39579775

[5] Drucker DJ . GLP-1-based therapies for diabetes, obesity and beyond. Nat Rev Drug Discov. (2025) 24:631–50. doi: 10.1038/s41573-025-01183-8. PMID: 40281304

[6] Shuai Y Yang G Zhang Q Li W Luo Y Ma J . Efficacy and safety of polyethylene glycol loxenatide monotherapy in type 2 diabetes patients: A multicentre, randomized, double-blind, placebo-controlled phase 3a clinical trial. Diabetes Obes Metab. (2021) 23:116–24. doi: 10.1111/dom.14198. PMID: 32965075

[7] Cai H Chen Q Duan Y Zhao Y Zhang X . Short-term effect of polyethylene glycol loxenatide on weight loss in overweight or obese patients with type 2 diabetes: An open-label, parallel-arm, randomized, metformin-controlled trial. Front Endocrinol (Lausanne). (2023) 14:1106868. doi: 10.3389/fendo.2023.1106868. PMID: 36777344 PMC9909427

[8] Liu Y Ma W Fu H Zhang Z Yin Y Wang Y . Efficacy of polyethylene glycol loxenatide for type 2 diabetes mellitus patients: a systematic review and meta-analysis. Front Pharmacol. (2024) 15:1235639. doi: 10.3389/fphar.2024.1235639. PMID: 38469407 PMC10925615

[9] Gao F Lv X Mo Z Ma J Zhang Q Yang G . Efficacy and safety of polyethylene glycol loxenatide as add-on to metformin in patients with type 2 diabetes: A multicentre, randomized, double-blind, placebo-controlled, phase 3b trial. Diabetes Obes Metab. (2020) 22:2375–83. doi: 10.1111/dom.14163. PMID: 32744358

[10] Sørensen KK Yazdanfard PDW Zareini B Pedersen-Bjergaard U Kosjerina V Andersen MP . Real-world cardiovascular effectiveness of sustained glucagon-like peptide 1 GLP-1 receptor agonist usage in type 2 diabetes. Cardiovasc Diabetol. (2025) 24:385. doi: 10.1186/s12933-025-02915-1. PMID: 41053738 PMC12502128

[11] Morales DR Arlett P . RCTs and real world evidence are complementary, not alternatives. Bmj. (2023) 381:736. doi: 10.1136/bmj.p736. PMID: 37011918

[12] Zhu J Han J Liu L Liu Y Xu W Li X . Clinical expert consensus on the assessment and protection of pancreatic islet beta-cell function in type 2 diabetes mellitus. Diabetes Res Clin Pract. (2023) 197:110568. doi: 10.1016/j.diabres.2023.110568. PMID: 36738836

[13] Battelino T Danne T Bergenstal RM Amiel SA Beck R Biester T . Clinical targets for continuous glucose monitoring data interpretation: Recommendations from the International Consensus on Time in Range. Diabetes Care. (2019) 42:1593–603. doi: 10.2337/dci19-0028. PMID: 31177185 PMC6973648

[14] Nauck MA Quast DR Wefers J Meier JJ . GLP-1 receptor agonists in the treatment of type 2 diabetes - state-of-the-art. Mol Metab. (2021) 46:101102. doi: 10.1016/j.molmet.2020.101102. PMID: 33068776 PMC8085572

[15] Chen Y Chen J Zhang S Zhu D Deng F Zuo R . Real-world effectiveness of GLP-1 receptor agonist-based treatment strategies on "time in range" in patients with type 2 diabetes. Front Pharmacol. (2024) 15:1370594. doi: 10.3389/fphar.2024.1370594. PMID: 38515845 PMC10955089

[16] Mohammedi K Belhatem N Berentzen TL Catarig AM Potier L . Once-weekly semaglutide use in patients with type 2 diabetes: Results from the SURE France multicentre, prospective, observational study. Diabetes Obes Metab. (2023) 25:1855–64. doi: 10.1111/dom.15045. PMID: 36869853

[17] Rajamand Ekberg N Bodholdt U Catarig AM Catrina SB Grau K Holmberg CN . Real-world use of once-weekly semaglutide in patients with type 2 diabetes: Results from the SURE Denmark/Sweden multicentre, prospective, observational study. Prim Care Diabetes. (2021) 15:871–8. doi: 10.1016/j.pcd.2021.06.008. PMID: 34183269

[18] Han J Lee WJ Hur KY Cho JH Lee BW Park CY . Safety and effectiveness of dulaglutide in the treatment of type 2 diabetes mellitus: A Korean real-world post-marketing study. Diabetes Metab J. (2024) 48:418–28. doi: 10.4093/dmj.2023.0030. PMID: 38310883 PMC11140407

[19] Jiang Y Bai HS Liu GX Wang SY Yin L Hou ZT . Effectiveness and safety of glucagon-like peptide 1 receptor agonists in patients with type 2 diabetes: evidence from a retrospective real-world study. Front Endocrinol (Lausanne). (2024) 15:1347684. doi: 10.3389/fendo.2024.1347684. PMID: 38524632 PMC10958196

[20] Xie C Alkhouri N Elfeki MA . Role of incretins and glucagon receptor agonists in metabolic dysfunction-associated steatotic liver disease: Opportunities and challenges. World J Hepatol. (2024) 16:731–50. doi: 10.4254/wjh.v16.i5.731. PMID: 38818288 PMC11135259

[21] Wong HJ Sim B Teo YH Teo YN Chan MY Yeo LLL . Efficacy of GLP-1 receptor agonists on weight loss, BMI, and waist circumference for patients with obesity or overweight: A systematic review, meta-analysis, and meta-regression of 47 randomized controlled trials. Diabetes Care. (2025) 48:292–300. doi: 10.2337/dc24-1678. PMID: 39841962

[22] Committee. A.D.A.P.P . 9. Pharmacologic approaches to glycemic treatment: Standards of care in diabetes-2025. Diabetes Care. (2025) 48:S181–s206. doi: 10.2337/dc25-S009. PMID: 39651989 PMC11635045

[23] Li J Tian Y Li L Zhao Y Yang S Xu W . Once-weekly glucagon-like peptide receptor agonist polyethylene glycol loxenatide protects against major adverse cardiovascular events in patients with type 2 diabetes: a multicenter ambispective cohort study (FLYING trial). MedComm (2020). (2025) 6:e70094. doi: 10.1002/mco2.70094. PMID: 39949982 PMC11822460

