# Supplementary material for: Efficacy of 12-week treatment with polyethylene glycol loxenatide in obesity or overweight patients with type 2 diabetes: a multicenter, prospective cohort study based on the flash glucose monitoring system
Source: Front Endocrinol (Lausanne). 2026 May 19;17:1810786. doi: 10.3389/fendo.2026.1810786 (PMC13226117; doi:10.3389/fendo.2026.1810786)
Supplement: Supplementary file 1 [file Table1.docx]

**Supplementary Table 1** Baseline characteristics of p included and excluded patients

| **Characteristics** | **Included (n=246)** | **Excluded (n=254)** | ***P*** |
| --- | --- | --- | --- |
| Sex (male/female) | 159/87 | 163/91 | 0.288 |
| Age (years) | 53.76 ± 10.86 | 48.32 ± 10.83 | 0.001 |
| Diabetic duration (years) | 4 (1, 10) | 5 (1, 11) | 0.413 |
| Weight (kg) | 75.92 ± 12.77 | 76.64 ± 14.32 | 0.122 |
| BMI (kg/m^2^) | 27.40 ± 3.46 | 27.86 ± 3.74 | 0.272 |
| WC (cm) | 95.50 ± 9.34 | 96.11 ± 9.25 | 0.241 |
| HbA_1c_ (%) | 8.74 ± 1.07 | 8.92 ± 1.32 | 0.670 |
| FBG (mmol/L) | 9.32±2.28 | 9.34 ± 2.47 | 0.722 |
| FC-p (ng/ml) | 2.17±1.03 | 2.15±1.04 | 0.548 |
| FINS (mU/L) | 8.91 (5.81, 12.97) | 9.22 (6.23, 14.93) | 0.187 |

BMI, body mass index; WC, waist circumference; FC-p, fasting C-peptide; FINS, fasting insulin.
